# Assessment of the association of the MOGAT1 and MOGAT3 gene with growth traits in different growth stages in Holstein calves

**DOI:** 10.5194/aab-65-301-2022

**Published:** 2022-08-10

**Authors:** Gökhan Gökçe, Mervan Bayraktar

**Affiliations:** Department of Animal Science, Çukurova University, Adana, Türkiye

## Abstract

The members of the monoacylglycerol acyltransferase (MOGAT) family are essential candidate genes that influence economic traits associated with triglyceride synthesis, dietary fat absorption, and storage in livestock. In addition, the MOGAT gene family may also play an essential function in human polygenic diseases, like type 2 diabetes and obesity. The present study was conducted on Holstein calves to find the association between MOGAT1, MOGAT3/g.A229G, and MOGAT3/g.G1627A and growth traits. The polymerase chain reaction–restriction fragment length polymorphism (PCR-RFLP) method was performed for genotyping the MOGAT1, MOGAT3/g.A229G, and MOGAT3/g.G1627A genes' locus using the *TaqI*, *MspI*, and *BsuRI* restriction enzyme. The allele frequency of A and G of the MOGAT1 locus was 0.79 and 0.21, respectively, while the genotype frequency was 0.65, 0.28, and 0.07 for AA, AG, and GG, respectively. While the allele and genotype frequencies of the MOGAT3/g.A229G locus were 00.57( $A_{1}$ ), 0.43( $G_{1}$ ), 0.35( $A_{1}A_{1}$ ), 0.45( $A_{1}G_{1}$ ), and 0.20( $G_{1}G_{1}$ ), the allele and genotype frequencies of the MOGAT3/g.G1627A locus were 0.49( $A_{2}$ ), 0.51( $G_{2}$ ), 0.25( $A_{2}A_{2}$ ), 0.49( $A_{2}G_{2}$ ), and 0.26( $G_{2}G_{2}$ ). Chi-square analysis showed that MOGAT3/g.G1627A distribution was at the Hardy–Weinberg disequilibrium ( p < 0.05), and MOGAT1 and MOGAT3/g.A229G distribution was at the Hardy–Weinberg equilibrium ( p > 0.05). In total, two statistical methods (general linear model (GLM) and PROC MIXED) were used to identify an association between gene locus and growth traits. An association analysis showed a statistically significant difference between the MOGAT1 and body weight, body length, and chest circumference, MOGAT3/g.A229G with average daily gain (ADG) and withers height, and MOGAT3/g.G1627A with body weight and body length ( p < 0.05). The results confirmed that the MOGAT1, MOGAT3/g.A229G, and MOGAT3/g.G1627A locus are strong candidate genes that could be considered molecular markers for growth traits in cattle breeding.

## 1 Introduction

Meat production depends mainly on the process of growth and development that occurs in the animal's body until it becomes appropriate for slaughter. Knowledge of the factors that affect growth is necessary to guide production processes for the welfare of animals (Szewczuk et al., 2013; te Pas, 2004; Zalewska et al., 2021). So far, not all aspects of the growth process are apparent. However, growth is known as the natural process of increasing in size, which results from the increased deposition of tissues similar in composition to the original tissue of the animal (Borges et al., 2014; Kowalczyk et al., 2022; Prihandini et al., 2019). This increase in volume is obtained through any of the following operations: increasing the size of cells without increasing their number (hypertrophy), increasing the number of cells without increasing their size (hyperplasia), and a cumulative increase in non-cellular components (Bayraktar and Shoshin, 2022a; Fadhil and Zülkadir, 2021; Pearson, 1990; Owens et al., 1993). A distinction must be made between true growth and fattening because true growth includes increasing the animal's size from structural tissues such as muscles and bones. In contrast, fattening increases size caused by increased fatty tissue (Bayraktar and Shoshin, 2022b; Jo et al., 2009). With the development of molecular techniques, it has become possible to identify genes that directly or indirectly affect the growth characteristics of animals (Bayraktar and Shoshin, 2022c; Park et al., 2018). Acyl coenzyme A, monoacylglycerol acyltransferase (MOGAT), also known as MGAT, is a vital enzyme that plays an active role in catalyzing the synthesis of triacylglycerol (TAG) and diacylglycerol (DAG; Lee and Kim, 2017; Cao et al., 2003; Hall et al., 2012). MOGAT acts to remove dietary fats (TAG) from the intestines by absorbing excess fats. MOGAT activity was found at high levels in the liver of suckling rats, diabetic animals, and the white adipose tissue of migratory birds and animals with a long hibernation period (Cheng et al., 2003; Yen et al., 2002; Mcfie et al., 2022; Schweitzer and Finck, 2014). The biochemical properties of MOGAT have been extensively investigated in the intestines, liver, kidney, and adipocytes of various types of animals (Yang and Nickels, 2015; Sankella et al., 2016). There are three different genes of MOGAT that have been identified in mammals, namely MOGAT1 (Yen et al., 2002), MOGAT2 (Cao et al., 2003; Yen and Farese, 2003), and MOGAT3 (Cheng et al., 2003). Monoacylglycerol O-acyltransferase 1 (MOGAT1) was first detected as a microsomal enzyme stimulating diacylglycerol (DAG) and TAG synthesis in mice (Winter et al., 2003). MOGAT2 has a vital function in absorbing dietary fats and is highly expressed in the small intestine. MOGAT3 is similar, in high-sequence homology, to diacylglycerol O-acyltransferase 2 (DGAT2) in humans and mammals and is not similar in rodents. All three MOGAT gene families are localized in the endoplasmic reticulum (ER) but differ in their expression patterns and catalytic properties in different tissues (Yang and Nickels, 2015). MOGAT1 plays an essential role in reducing obesity and hepatic steatosis. Little is known about the function of MOGAT1 in the cell. However, MOGAT1 has an essential role in reducing obesity and hepatic steatosis (Sun et al., 2012). Lee and Kim (2017) detected interaction MOGAT1 with DGAT2, which increases the synergy of the TAG biosynthesis and lipid droplets expansion, leading to an increase in lipid accumulation in the liver and fat. Bovine MOGAT1 is located on chromosome 2 (BTA7) with six exons and five introns. The MOGAT1 gene has 34 976 bp , 1 transcript (splice variant), 220 orthologues, and 9 paralogues (NC_037329.1, ENSBTAG00000008431). Studies have shown the effect of MOGAT1 on growth and milk fatty acid traits in cattle breeds (Shi et al., 2019; Lyu et al., 2021). MOGAT3 is expressed in the liver, adipose tissue, and testes. MOGAT3 is believed to have a role in lipid metabolism; however, little is known about its physiological functions. When DAGs or monoacylglycerols (MAGs) are used as substrates, MOGAT3 shows higher DGAT activity than MOGAT1 and MOGAT2 (Yang and Nickels, 2015). Bovine MOGAT3 is mapped on chromosome 25 (BTA25), with eight exons and six introns. The MOGAT3 gene has 5682 bp , and the transcript length is 1026 bp . This study aims to estimate the polymorphisms of MOGAT1 and MOGAT3 and detect these genes' effects on growth traits in Holstein calves.

## 2 Materials and methods

### 2.1 Experimental animals, data collection, and genomic DNA extraction

A total of 101 Holstein calves (male and female) were collected from the Çukurova University Research and Application Farm, Adana, Türkiye. Calves were fed ad libitum with a starter feed and alfalfa. Body measurement traits (body weight, body length, withers height, chest circumference, and shank circumference) were measured at birth, 1 months, 2 months, and weaning. The average daily gain (ADG) was calculated from birth to weaning. DNA was extracted from blood samples collected from the jugular vein and stored at - 20 ${}^{\circ }C$ according to the salting-out procedure, with slight modifications (Miller et al., 1988). DNA content was calculated by spectrophotometer. DNA samples were stored at - 20 ${}^{\circ }C$ until subsequent analysis.

**Table 1:** The MOGAT1 and MOGAT3 primer sequence and PCR condition.

Locus	Primer sequencing	PCR	Restrictionenzyme	PCRcondition	Reference
MOGAT1	5 ${}^{\prime }$ -CGAGTTTGCGCCACTCAACA-3 ${}^{\prime }$ 5 ${}^{\prime }$ -GGAACACGATAATTCCGAGGC-3 ${}^{\prime }$	461 bp	*TaqI*	95 ${}^{\circ }C$ at 5 m ,94 ${}^{\circ }C$ at 30 s ,59 ${}^{\circ }C$ at 40 s ,72 ${}^{\circ }C$ at 40 s ,36 cycles of72 ${}^{\circ }C$ at 10 m	Lyu et al. (2021)
MOGAT3/g.A229G	5 ${}^{\prime }$ -AGGCACCTCGTCTTTATCT-3 ${}^{\prime }$ 5 ${}^{\prime }$ -CCTTACCCAGGAAGAGGAAAC-3 ${}^{\prime }$	668 bp	*MspI*	94 ${}^{\circ }C$ at 5 m ,94 ${}^{\circ }C$ at 45 s ,62 ${}^{\circ }C$ at 1 m ,72 ${}^{\circ }C$ at 45 s ,32 cycles of72 ${}^{\circ }C$ at 10 m	Sun et al. (2012)
MOGAT3/g.G1627A	5 ${}^{\prime }$ -CTGTTCCCAGGGCTTCGGTT-3 ${}^{\prime }$ 5 ${}^{\prime }$ -CCAATTCAAGCCAAGTGCCTG-3 ${}^{\prime }$	180 bp	*BsuRI*	94 ${}^{\circ }C$ at 5 m ,94 ${}^{\circ }C$ at 45 s ,66 ${}^{\circ }C$ at 45 s ,72 ${}^{\circ }C$ at 45 s ,32 cycles of72 ${}^{\circ }C$ at 10 m	

### 2.2 PCR amplification and genotyping

The PCR conditions and primer sequences of the MOGAT1 and MOGAT3 locus are shown in Table 1. The PCR reaction was performed in a 20 µL volume, containing 5 µL (50 ng ) DNA, 5 µL of PCR Master Mix (Thermo Scientific, USA), 0.5 µL for each primer (forward and reverse), and 9 µL of distilled water. The PCR products were digested by *TaqI*, *MspI*, and *BsuRI* FastDigest Restriction Enzymes at 37 ${}^{\circ }C$ for 5–15 min (Thermo Scientific, USA). The reaction content was as follows: PCR products (8 µL ), distilled water (4 µL ), 10X buffer (2 µL ), and restriction enzymes (1 µL ). The digested product was separated in 2 % agarose gel and stained with ethidium bromide. The fragment length was visualized using an imaging system.

### 2.3 Statistical analysis

The allele and genotype frequencies of the MOGAT1 and MOGAT3 gene locus and gene distribution according to the Hardy–Weinberg equilibrium were calculated by POPGENE software.

### 2.4 Association analysis

In total, two statistical methods were used to estimate the association between gene polymorphism and growth traits. The general linear model (GLM) was used to determine the associations between gene polymorphism and the ADG. A mathematical linear model was applied as follows:

$$Y_{ij}=\mu +\alpha_{i}+\beta_{j}+E_{ij},$$

where $Y_{ij}$ is the phenotypic value, μ is the overall means, $\alpha_{i}$ is the effect of genotypes, $\beta_{j}$ is the effect of sex (male and female), and $E_{ij}$ is the random errors.

The repeated measures using the PROC MIXED procedure by SAS was used to estimate the association between gene polymorphism and growth traits. The model was applied as follows:

$$Y_{ijk}=\mu +\alpha_{i}+\beta_{j}+G_{k}+E_{ijk},$$

where $Y_{ijk}$ is the phenotypic value, μ is the overall means, $\alpha_{i}$ is the fixed effect of i th genotypes, $\beta_{j}$ is the fixed effect of j th sex (male and female), $G_{k}$ is the fixed effect of k th time of measurement traits, and $E_{ijk}$ is the random error.

The restricted maximum likelihood (REML) approach was used for unbiased calculations of covariance structure. The Akaike information criterion (AIC) was used to select the most appropriate covariance structure for the data. In the selection of this criterion, it is aimed to have the lowest type 1 error. Autoregressive (ar1) was used as covariance structure in the analysis.

**Figure 1:**
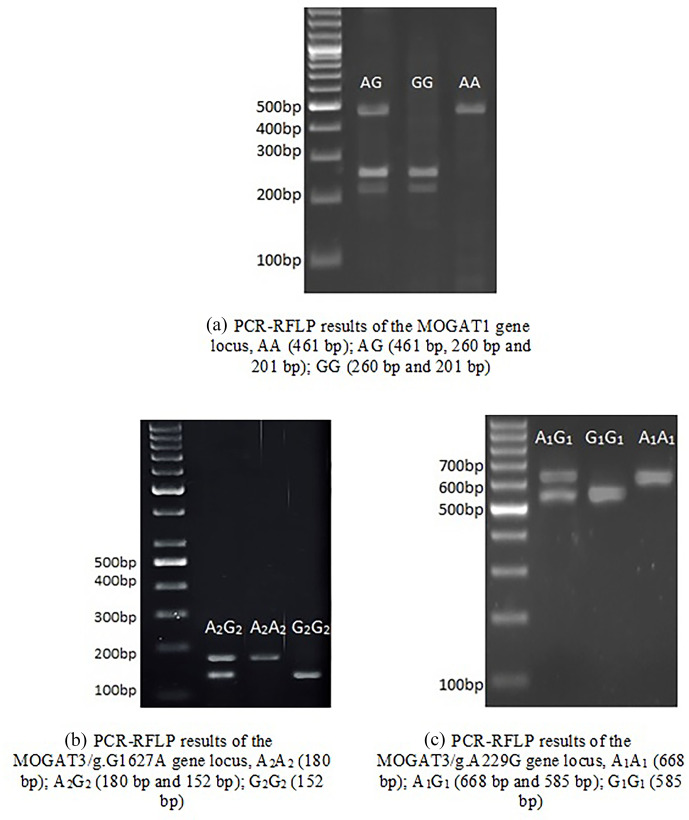
Agarose gel electrophoresis patterns.

**Table 2:** Genotype and allele frequencies of the MOGAT1, MOGAT3/g.A229G, and MOGAT3/g.G1627A.

Gene locus	Genotype frequency	Allele frequency	$\chi^{2}$ (HWE)
MOGAT1	AA(0.65)AG(0.28)GG(0.07)	A(0.79)G(0.21)	2.43
MOGAT3/g.A229G	$A_{1}A_{1}$ (0.35) $A_{1}G_{1}$ (0.45) $G_{1}G_{1}$ (0.20)	$A_{1}$ (0.57) $G_{1}$ (0.43)	0.62
MOGAT3/g.G1627A	$A_{2}A_{2}$ (0.25) $A_{2}G_{2}$ (0.49) $G_{2}G_{2}$ (0.26)	$A_{2}$ (0.49) $G_{2}$ (0.51)	0.04 ${}^{*}$

$\chi^{2}$ is chi-square, HWE is the Hardy–Weinberg equilibrium, and ${}^{*}$ p < 0.05.

## 3 Results

### 3.1 MOGAT1 gene polymorphism

In total, 461 bp of PCR products were amplified. There were three genotypes (AA, AG, and GG) observed after digesting PCR products by *TaqI*. The fragment lengths are as follows: AA 461 bp , AG 461, 260, and 201 bp , and GG 260 and 201 bp (Fig. 1). $\chi^{2}$ tests were not significant ( p > 0.05). The genotype and allele frequency were 0.65(AA), 0.28(AG), 0.007(GG), 0.79(A), and 0.21(G) (Table 2).

### 3.2 MOGAT3/g.A229G gene polymorphism

There were three genotypes ( $A_{1}A_{1}$ , $A_{1}G_{1}$ , and $G_{1}G_{1}$ ) identified after digesting 668 bp of PCR products. The fragment lengths are as follows: $A_{1}A_{1}$ 668 bp , $A_{1}G_{1}$ 668 and 585 bp , and $G_{1}G_{1}$ 585 and 83 bp (because of the small size of the 83 bp fragment, it did not appear in the gel). $\chi^{2}$ tests were not significant ( p > 0.05). The genotype and allele frequency were 0.35( $A_{1}A_{1}$ ), 0.45( $A_{1}G_{1}$ ), 0.20( $G_{1}G_{1}$ ), 0.57( $A_{1}$ ), and 0.43( $G_{1}$ ) (Table 2).

### 3.3 MOGAT3/g.G1627A gene polymorphism

The 180 bp PCR products were digested with *BsuRI*, and three genotypes ( $A_{2}A_{2}$ , $A_{2}G_{2}$ , and $G_{2}G_{2}$ ) were identified. The fragment lengths are as follows: $A_{2}A_{2}$ 180 bp $A_{2}G_{2}$ 180 and 152 bp , and $G_{2}G_{2}$ 152 and 28 bp . $\chi^{2}$ tests were significant ( p < 0.05). The genotype and allele frequency were 0.25( $A_{2}A_{2}$ ), 0.49( $A_{2}G_{2}$ ), 0.26( $G_{2}G_{2}$ ), 0.49( $A_{2}$ ), and 0.51( $G_{2}$ ) (Table 2).

**Table 3:** Association analysis of genotypes and growth traits according PROC MIXED.

Traits	Genotypes			p
	MOGAT1			
	AA	AG	GG	
Body weight ( kg )	52.65 ± 0.54 ${}^{b}$	54.26 ± 0.40 ${}^{a}$	56.57 ± 0.51 ${}^{a}$	0.022 ${}^{*}$
Body length ( cm )	75.05 ± 0.42 ${}^{b}$	76.45 ± 0.31 ${}^{a}$	78.68 ± 0.39 ${}^{a}$	0.043 ${}^{*}$
Chest circumference ( cm )	80.33 ± 0.43 ${}^{b}$	81.74 ± 0.31 ${}^{a}$	83.96 ± 0.40 ${}^{a}$	0.010 ${}^{*}$
Withers height ( cm )	81.89 ± 0.39	85.41 ± 0.28	83.61 ± 0.36	0.251
Shank circumference ( cm )	10.58 ± 0.05	10.75 ± 0.03	11.78 ± 0.05	0.394
	MOGAT3/g.A229G			
	$A_{1}A_{1}$	$A_{1}G_{1}$	$G_{1}G_{1}$	
Body weight ( kg )	54.16 ± 0.53	55.13 ± 0.48	54.09 ± 0.59	0.276
Body length ( cm )	76.41 ± 0.40	75.47 ± 0.36	76.30 ± 0.44	0.167
Chest circumference ( cm )	81.69 ± 0.41	80.79 ± 0.37	81.55 ± 0.45	0.211
Withers height ( cm )	83.27 ± 0.36 ${}^{a}$	82.46 ± 0.33 ${}^{b}$	85.59 ± 0.40 ${}^{b}$	0.032 ${}^{*}$
Shank circumference ( cm )	10.75 ± 0.05	10.63 ± 0.04	10.73 ± 0.05	0.183
	MOGAT3/g.G1627A			
	$A_{2}A_{2}$	$A_{2}G_{2}$	$G_{2}G_{2}$	
Body weight ( kg )	52.47 ± 0.55 ${}^{b}$	56.58 ± 0.44 ${}^{a}$	54.43 ± 0.61 ${}^{a}$	0.015 ${}^{*}$
Body length ( cm )	75.23 ± 0.41 ${}^{b}$	77.58 ± 0.33 ${}^{a}$	75.52 ± 0.46 ${}^{a}$	0.013 ${}^{*}$
Chest circumference ( cm )	80.50 ± 0.42	81.63 ± 0.34	81.90 ± 0.47	0.078
Withers height ( cm )	82.27 ± 0.37	84.35 ± 0.30	83.29 ± 0.42	0.069
Shank circumference ( cm )	10.60 ± 0.05	10.74 ± 0.04	10.77 ± 0.05	0.088

${}^{*}$ p < 0.05

**Table 4:** Association analysis of genotypes and ADG according GLM.

Trait	Genotypes			p
ADG	MOGAT1			
	AA	AG	GG	
	71.871 ± 0.627	72.437 ± 0.507	72.909 ± 0.662	0.516
	MOGAT3/g.A229G			
	$A_{1}A_{1}$	$A_{1}G_{1}$	$G_{1}G_{1}$	
	71.224 ± 0.604 ${}^{b}$	72.323 ± 0.490 ${}^{\mathrm{ab}}$	74.671 ± 0.716 ${}^{a}$	0.040 ${}^{*}$
	MOGAT3/g.G1627A			
	$A_{2}A_{2}$	$A_{2}G_{2}$	$G_{2}G_{2}$	
	73.081 ± 0.611	73.929 ± 0.517	72.209 ± 0.675	0.336

ADG is average daily gains; ${}^{*}$ p < 0.05

### 3.4 Association analysis between genotypes and growth traits

The association results showed a significant association between growth traits and calves. MOGAT1 showed an association with body weight, body length, and chest circumference ( p < 0.05), with no association with withers height and shank circumference (Table 3). The means of body weight, body length, and chest circumference for genotypes were as follows: AA (52.65 ± 0.54), AG (54.26 ± 0.40), and GG (55.57 ± 0.51), AA (75.05 ± 0.42), AG (76.45 ± 0.31), and GG (77.68 ± 0.39), and AA (80.33 ± 0.4294), AG (81.74 ± 0.31), and GG (83.96 ± 0.40), respectively. The MOGAT3/g.A229G showed significant association with withers height and no association with other traits. The means of withers height for $A_{1}A_{1}$ , $A_{1}G_{1}$ and $G_{1}G_{1}$ were (83.27 ± 0.36), (82.46 ± 0.33) and (84.59 ± 0.40), respectively. The MOGAT3/g.G1627A showed an association with body weight and body length. The means of body weight and body length for $A_{2}A_{2}$ , $A_{2}G_{2}$ and $G_{2}G_{2}$ were as follows: (52.47 ± 0.55), (54.18 ± 0.44), and (55.73 ± 0.61) and (75.23 ± 0.41), (75.38 ± 0.33), and (76.57 ± 0.46), respectively. According to the ADG, a significant association was found between MOGAT3/g.A229G and the ADG, where the means of the $A_{1}A_{1}$ , $A_{1}G_{1}$ and $G_{1}G_{1}$ were as follows: (71.224 ± 0.604), (72.323 ± 0.490), and (74.671 ± 0.716), respectively (Table 4).

## 4 Discussion

Recently, genetic association studies have become of great interest to researchers, especially for discovering novel candidate genes that influence economic traits in farm animals. The genes of the MOGAT family were recently studied in cattle breeds. However, the association studies in this regard are limited. The MOGAT family members (MOGAT1, MOGAT2, and MOGAT3) affect the metabolism, absorption, and storage of fats (Yang and Nickels, 2015; Agarwal et al., 2016; Soufi et al., 2014). This study determined the association of MOGAT1 and MOGAT3 gene locus with growth traits in Holstein calves. There were three genotypes, AA, AG, and GG, identified within the MOGAT1 gene. The g.25940T > C locus of the MOGAT1 gene was observed in intron 5. The allele frequencies of A and G were 0.79 and 0.21, respectively. Also, the A allele frequency was higher than G allele in native Chinese cattle breeds, where the A allele frequency in the Chinese breeds was 0.96, 0.70, 0.85, and 0.95 (Lyu et al., 2021). The association analysis showed a significant association between MOGAT1 locus and body weight, body length, and chest circumference. The GG genotype had a significantly higher body weight (56.57 ± 0.51), body length (78.68 ± 0.39), and chest circumference (83.96 ± 0.40) compared to the AA and AG genotypes. A similar trend was identified in Qingchuan and Xianan cattle, where animals with GG genotypes had higher body weight compared to other genotypes (Lyu et al., 2021). In contrast, the animals with AG genotype had a higher chest depth than other genotypes in Yunling cattle. Shi et al. (2019) observed two single nucleotide polymorphisms, g.111599360A > G and g.111601747 T > A, of the MOGAT1 gene and their effects on milk fatty acid traits in Chinese Holstein. Winter et al. (2003) determined an association between MOGAT1 and milk fat composition in the German dairy breeds and also confirmed the substitution of cysteine (Cys) with lysine (Lys) due to a missense mutation in exon 4. Mutations in MOGAT1 are believed to change promoter activities (Shi et al., 2019). There were three genotypes observed at the MOGAT3/g.A229G locus. The allele and genotype frequency were $A_{1}A_{1}$ (0.35), $A_{1}G_{1}$ (0.45), $G_{1}G_{1}$ (0.20) and $A_{1}$ (0.57) and $G_{1}$ (0.43). Sun et al. (2012) showed $A_{1}$ allele frequency between 0.49 and 0.83 and $G_{1}$ frequency between 0.167 and 0.502 in Luxi, Nanyang, Jiaxian, Caoyuan, and Qinchuan native Chinese cattle breeds. It showed that the $A_{1}$ allele frequency of Holstein calves in the current study is in agreement with the frequency of Luxi, Nanyang, and Jiaxian. Association analysis showed a significant association between MOGAT3/g.A229G locus and ADG and withers height trait. The $G_{1}G_{1}$ genotype showed a higher means of ADG and withers height from $A_{1}A_{1}$ and $A_{1}G_{1}$ . Also, the $G_{1}G_{1}$ genotype showed a higher means of withers height than other genotypes in Nanyang cattle. In the same study, the $A_{1}G_{1}$ genotype showed a higher means of body mass and ADG (Sun et al., 2012). There were three genotypes determined at the MOGAT3/g.G1627A locus. The genotype and allele frequency were $A_{2}A_{2}$ (0.25), $A_{2}G_{2}$ (0.49), and $G_{2}G_{2}$ (0.26) and $A_{2}$ (0.49) and $G_{2}$ (0.51). Chi-square analysis showed the MOGAT3/g.G1627A loci at the Hardy–Weinberg disequilibrium ( p < 0.05). Sun et al. (2012) observed $A_{2}$ allele frequency between 0.403 and 0.675 and $G_{2}$ allele frequency between 0.32 and 0.59 in five Chinese cattle breeds. Our results in terms of A2 and G2 allele frequency were in agreement with A2(0.470) and G2(0.530) in Jiaxian cattle breeds. The $A_{2}G_{2}$ genotype was observed to have a significant value for the body weight and body length compared to $A_{2}A_{2}$ and $G_{2}G_{2}$ . Also, the $A_{2}G_{2}$ genotype showed higher means for body mass, ADG, and hucklebone width than other genotypes in Nanyang cattle breeds (Sun et al., 2012). The two loci g.A229G and g.G1627A were identified in exon 1 and 4 of the bovine MOGAT3 gene, as these mutations change the amino acid sequence, leading to a change in the protein's function.

## 5 Conclusion

This study determined a significant association between MOGAT1 and MOGAT3 genes and growth traits in Holstein calves. The Holstein calves showed polymorphisms of the MOGAT1 and MOGAT3 gene locus. The AA genotype for MOGAT1, $A_{1}G_{1}$ for MOGAT3/g.A229G, and $A_{2}G_{2}$ for MOGAT3/g.G1627A were the most common in the studied sample. The animals with a GG genotype of MOGAT1 outperformed the other genotypes in body weight, body length, and chest circumference, while the $G_{1}G_{1}$ genotype of MOGAT3/g.A229G showed the highest value of ADG and withers height. Also, the $A_{2}G_{2}$ genotype of MOGAT3/g.G1627A had the most body weight and body length of other genotypes. In conclusion, the MOGAT1 and MOGAT3 locus may be used as molecular marker in animal improvement programs.

## Data Availability

The data will be made available from the corresponding author upon request.
